# Adolescents and young adults with TB in a low-incidence setting

**DOI:** 10.5588/ijtldopen.25.0031

**Published:** 2025-06-13

**Authors:** A. Duret, A. Cardoso-Pinto, A. Bhattacharyya, Ivin Jose, A. Ahmadi, I. Patton, A. Ostrzewska, H. Durkan, O.M. Kon, J.A. Seddon, E. Whittaker

**Affiliations:** ^1^ Department of Paediatric Infectious Diseases, Imperial College Healthcare NHS Trust, London, UK;; ^2^ Department of Paediatrics, University of Leicester NHS Trust, Leicester, UK;; ^3^ Department of Infectious Diseases, Imperial College Healthcare NHS Trust, London, UK;; ^4^ Desmond Tutu TB Centre, Department of Paediatrics and Child Health, Stellenbosch University, Cape Town, South Africa.

**Keywords:** tuberculosis, United Kingdom, adolescent health, young adults, cavitary disease, social risk factors

## Abstract

**BACKGROUND:**

Adolescents and young adults (AYA) with TB have distinct physical and social characteristics compared to other age groups. This study describes a cohort of AYA with TB in a low TB-prevalence, high-income setting and investigates whether demographic or social factors affect management outcomes.

**METHODS:**

A retrospective cohort study was conducted at a TB referral centre in North West London, including patients aged 10–24 years from 2015 to 2022. Median days from symptom onset to healthcare presentation were determined and risk factors for late presentation (>60 days) were assessed.

**RESULTS:**

Among 158 patients (median age 20 years, IQR 17–23), 53.6% had pulmonary TB, 39.9% extrapulmonary disease, and 5.7% disseminated disease; 25.3% had cavities. Social risk factors were present in 32.3% of patients. Median delay to presentation was 45 days (IQR 14–96), with 38.7% presenting after two months. Delays were longer in patients with incarceration, drug misuse, or mental health issues, though not statistically significant. Patients with social risk factors were more likely to receive observed therapy (OR 2.65, IQR 1.27–5.64).

**CONCLUSIONS:**

AYA with TB in this setting experienced delays in healthcare presentation and a quarter had cavitary disease. Social risk factors were common but not significantly related to outcomes.

TB follows a different clinical trajectory in adolescents compared to younger children. Whereas the latter often present with paucibacillary TB,^1^ adolescents are more susceptible to adult-like disease patterns, with more severe pulmonary involvement.^1–3^ Physiological and immunological changes during adolescence have been hypothesised to contribute to this shift.^1^ The pulmonary manifestations adolescents experience correlate with cavity formation and higher infectivity,^4^ as well as potentially increased risk of post-TB lung disease.^5,6^ Adolescence is a period of growing autonomy, with individuals increasingly taking responsibility for their health; however, this process is gradual, and adolescents still require support from healthcare providers, family and social systems.^7^ Adolescents and young adults (AYA) with TB often face substantial life challenges, including poverty, homelessness, substance abuse, mental health issues and recent immigration into the country.^8,9^ These AYA may already be experiencing disrupted education, unstable family environments and lack of social support,^10,11^ thus facing high barriers to accessing healthcare. Social determinants of health could therefore impact an individual’s ability to be assessed promptly after TB symptom onset, delay treatment initiation and hinder the achievement of positive outcomes.^8^

The United Kingdom (UK) has a low TB burden, with a national incidence of 8.5 per 100,000 in 2023.^12^ However, North West London has a higher incidence than the national average, with three local authorities (Brent, Ealing and Harrow) having TB notification rates of between 30 to 40 per 100,000, making them the districts with the third, fourth and fifth highest rates nationally.^12^ In children under 18 years old, the national TB notification rate was 2.2 per 100,000;^13^ 39.4% of children with TB were born in the UK, 9.6% had experienced homelessness and 20.5% were asylum-seekers.^13^ Many of these AYA are at higher risk of not completing TB treatment; observed therapy is an important resource to support these patients and was offered to nearly 30% of children and young people in 2023.^13^

We therefore set out to review all AYA with TB between 10–24 years old notified to the UK Health and Safety Agency (UKHSA, previously Public Health England) from a tertiary centre in North West London, between 2015 to 2022. As noted above, this region has a higher incidence and hence should capture a higher number of individuals with TB. We distinguished three age groups: young adolescents (10–14 years), older adolescents (15–19 years) and young adults (20–24 years). Here, we describe this cohort and investigate if social risk factors (SRF) or demographic factors are associated with delayed presentation to healthcare, receipt of observed therapy or cavitary disease.

## METHODS

### Study design and context

All individuals with TB between 10–24 years notified to the London TB Register between 1st January 2015 to 31st December 2022 by one North West London National Health Service (NHS) Trust were considered.^14^ These dates coincided with the Trust’s introduction of electronic medical records, while the end date was set to have a final treatment outcome available for all patients. To be notified with TB, patients must either have culture confirmation of *M. tuberculosis* complex, or clinical or radiological signs of TB with initiation of anti-TB therapy; this notification is statutory.^14^ Patients under 16 are looked after by the Paediatric Infectious Disease team, while patients over 16 receive care from the Adult Respiratory team; the same team of TB clinical nurse specialists manage care for both age groups.

### Data collection and analysis

Clinical records were retrospectively reviewed, and demographic and clinical data were extracted into a data collection tool modelled on the national Enhanced TB Surveillance system database.^15^ All data analysis was performed in R v4.4. Continuous variables were summarised using medians and interquartile ranges (IQRs); categorical variables were summarised using frequencies and percentage of total patient group. Age was converted into a categorical variable, using the three groups described above. To investigate associations between SRFs, demographic factors and delayed presentation to healthcare or cavitary disease, univariate analyses were performed to generate odds ratios (OR) and 95% confidence intervals (CI).

This study was approved by the HRA and Health and Care Research Wales Research Ethics Committee (REC reference 22/WA/0221).

## RESULTS

A total of 158 patients between 10–24 years of age were notified to have TB by the study centre between 2015 to 2022. The median age was 20 years (IQR 17–23), with 95 (60.1%) males. Sixty-nine (43.7%) were born in low-incidence countries, with 89 (56.3%) born in a country with a high incidence of TB (≥40 per 100,000 as per UKHSA).^16^ Younger adolescents were more likely to be born in low-incidence countries than other age groups, and young adults more likely to be born in high-incidence countries than other age groups (Fisher’s exact test p=0.003 for both), see Table 1. Most patients were immunocompetent, with 5 (3.2%) patients living with HIV. Eighty-five patients (53.8%) had pulmonary TB (PTB) with similar proportions across all three age groups (Table 1). Only 9 patients (5.7%) had disseminated TB. Cavitary disease was present in 40 patients (47% of those with pulmonary disease). Microbiological confirmation by either polymerase chain reaction (PCR), microscopy or culture was achieved in 101 (63.9%) patients. Of these, 7 (5.1%) had multidrug-resistant TB (MDR-TB), defined as disease caused by resistance to at least isoniazid and rifampicin.^17^

**Table 1.** Summary of demographic and clinical characteristics of adolescents and young adults (AYA) with TB, by age group (10–14 years, 15–19 years and 20–24 years).

		10–14 years	15–19 years	20–24 years	Total
Age		(n = 20)	(n = 50)	(n = 88)	(n = 158)
Sex	Male	12 (60.0%)	29 (58.0%)	58 (65.9%)	95 (60.1%)
	Female	8 (40.0%)	21 (42.0%)	30 (34.1%)	63 (39.9%)
Birth country	Low incidence	15 (75.0%)	25 (50.0%)	29 (33.0%)	69 (43.7%)
	High incidence	5 (25.0%)	25 (50.0%)	59 (67.0%)	89 (56.3%)
Years since UK entry		7.5 (IQR 3–11)	2.0 (IQR 0–9.75)	4.0 (IQR 1–8)	4 (IQR 1–9.25)
BCG vaccination	Vaccinated	16 (80.0%)	20 (40.0%)	34 (38.6%)	70 (44.3%)
	Unvaccinated	1 (5.0%)	6 (12.0%)	1 (1.1%)	25 (15.8%)
	Unknown	3 (15.0%)	24 (48.0%)	35 (39.8%)	63 (39.9%)
Smoking status	Smoker	0 (0.0%)	5 (10.0%)	18 (20.5%)	23 (14.6%)
	Non-smoker	17 (85.0%)	36 (72.0%)	60 (68.2%)	113 (71.5%)
	Unknown	3 (15.0%)	9 (18.0%)	10 (11.4%)	22 (13.9%)
Employment status	Education	20 (100.0%)	38 (76.0%)	21 (23.9%)	79 (50.0%)
	Employed	0 (0.0%)	5 (10.0%)	41 (46.6%)	46 (29.1%)
	Unemployed	0 (0.0%)	1 (2.0%)	12 (13.6%)	13 (8.2%)
	Asylum seeker	0 (0.0%)	5 (10.0%)	6 (6.8%)	11 (7.0%)
	Incarcerated	0 (0.0%)	0 (0.0%)	4 (4.5%)	4 (2.5%)
	Unknown	0 (0.0%)	1 (2.0%)	2 (2.3%)	3 (1.9%)
Pulmonary TB	Total	11 (55.0%)	29 (58.0%)	45 (51.1%)	85 (53.8%)
	Cavitary disease	7 (35.0%)	13 (26.0%)	20 (22.7%)	40 (25.3%)
Extrapulmonary TB	Total	6 (30.0%)	17 (34.0%)	40 (45.5%)	63 (39.9%)
	Lymph nodes	2 (10.0%)	9 (18.0%)	16 (18.2%)	27 (17.1%)
	Gastrointestinal/peritoneal	1 (5.0%)	2 (4.0%)	5 (5.7%)	8 (5.1%)
	CNS	0 (0.0%)	1 (2.0%)	6 (6.8%)	7 (4.4%)
	Osteoarticular	1 (5.0%)	0 (0.0%)	6 (6.8%)	7 (4.4%)
	Ocular	0 (0.0%)	3 (6.0%)	4 (4.5%)	7 (4.4%)
	Pleural	0 (0.0%)	1 (2.0%)	2 (2.3%)	3 (1.9%)
Disseminated		3 (15.0%)	3 (6.0%)	3 (3.4%)	9 (5.7%)
Microbiological confirmation^A^		10 (50.0%)	33 (66.0%)	58 (65.9%)	101 (63.9%)

A polymerase chain reaction (PCR) positive for *M. tuberculosis* complex, or *M. tuberculosis* cultured from clinical sample.

### Delays in treatment initiation

**Figure 1.**
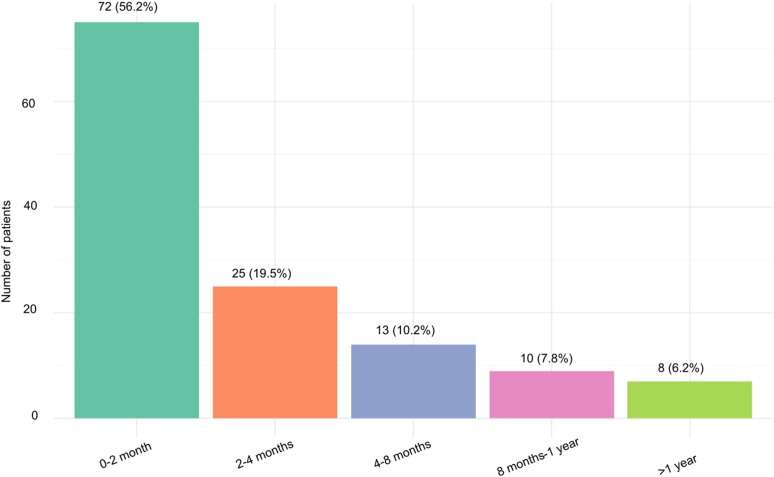
Delay between symptom onset and presentation to secondary healthcare.

The median time between TB symptom onset and secondary healthcare presentation was 45 days (IQR 14–96 days). Fifty-five (38.7%, n=142) presented to healthcare at least two months after symptom onset (Figure 1). The dates of symptom onset were not available for 16 patients. The following SRFs were considered: asylum-seeking status at time of diagnosis, mental health issues (either preceding TB diagnosis or developed while receiving TB care), and homelessness, incarceration, alcohol or drug misuse at any point. Of all patients, 51 (32.3%) had at least one SRF. No associations were observed between having one or more SRFs and presenting >30 days after symptoms onset (Table 2). To investigate factors that may be contributing to the range of delay between symptoms onset and presentation to healthcare, the OR of late presentation (>60 days from symptoms onset) in the presence of demographic (age and sex), clinical (cavitary disease) or SRFs were calculated. No statistically significant associations were identified (Table 2).

**Table 2.** Social risk factors prevalence in adolescents and young adults (AYA) with TB and median number of days from TB symptoms onset to presentation to healthcare.

Social risk factors (n = 158)			Days from TB symptoms onset to presentation to healthcare (median, IQR)	Mann-Whitney U test (p-value)
Any	51 (32.3%)	Yes	45 (19–90)	0.953
		No	44 (13–133)	
Homeless	26 (16.4%)	Yes	37 (10–72)	0.391
		No	47 (15–97)	
Asylum seeker	17 (10.8%)	Yes	28 (10–55)	0.266
		No	38 (14–105)	
Mental health	16 (10.1%)	Yes	62 (15–270)	0.446
		No	45 (14–86)	
Drug misuse	14 (8.9%)	Yes	147 (13–283)	0.221
		No	45 (15–86)	
Incarceration	9 (5.7%)	Yes	114 (45–273)	0.155
		No	43 (14–87)	
Alcohol misuse	5 (3.1%)	Yes	42 (9–138)	0.899
		No	45 (15–96)	

**Table 3.** Odds ratio for late presentation to healthcare (>60 days after symptoms onset) or cavitary disease based on demographic and social factors.

Late presentation		OR (95% CI) - univariate analysis
Age		1.06 (0.96–1.16)
Sex	Male	1.19 (0.58–2.45)
Social risk factors	Any	1.14 (0.54–2.42)

Cavitary disease		OR (95% CI) - univariate analysis
Age		0.94 (0.86–1.04)
Sex	Male	0.75 (0.36–1.57)
Social risk factors	Any	0.72 (0.31–1.56)
Late presentation		0.46 (0.19–1.05)

### TB treatment

TB treatment was recorded as completed in 135 (85.4%) patients. Twenty (12.7%) individuals were recorded as not completing treatment, including 5 who moved abroad before treatment completion. Only 3 patients were recorded as lost to follow-up with no handover of care. The use of observed therapy was recorded in 49 (31.0%) patients – those with at least one SRF were more likely to receive observed therapy (OR 2.65, 95%CI: 1.27–5.64). Most patients did not report severe treatment-related adverse events, with 35 patients (22.1%) experiencing at least one side effect; this was not associated with sex, age or SRFs. The most reported side effects were nausea and vomiting (15, 9.5%), followed by drug-induced liver injury (10, 6.3%). All patients were alive at the last recorded treatment outcome.

## DISCUSSION

To our knowledge, this the most extensive observational investigation to date of TB in adolescents within a high-income, low TB-prevalence country. Our study included 158 patients – for context, 1,331 patients <15 years and 37,318 patients ≥15 years were reported nationally over the same period.^18^ Our findings align with previous studies on adolescent TB, where PTB was notably more common than extrapulmonary TB.^1^ Strikingly, nearly half of patients with PTB presented with cavitary disease. There sparse published research to contextualise our findings, however one retrospective cohort study in Iran found that 29% of adolescents with PTB had cavities, in line with our study.^19^ This observation has important clinical implications, particularly concerning post-TB lung disease in adolescents. Cavitary TB, characterised by extensive lung damage and large cavities in the lung tissue, is associated with higher bacterial loads, increased transmissibility, and greater likelihood of long-term pulmonary complications.^20,21^ Several hypotheses exist regarding underlying reasons for higher prevalence of cavitary disease in adolescents. Adolescents may mount a more vigorous immune response to M. tb compared to younger children, leading to more pronounced inflammatory lung damage.^22,23^ During adolescence, substantial physiological changes, including hormonal shifts and evolving immune system functions, could alter the body's response to infections from pathogens like M. tb.^23^ Furthermore, factors such as delayed diagnosis, undernutrition, or coexisting medical conditions may contribute to the higher occurrence of cavitary disease in this population.^5^ Further research is needed to explore these hypotheses and elucidate the mechanisms driving cavitary TB in adolescents, which could help shape targeted interventions to prevent long-term lung damage in these patients.

Younger adolescents were more likely to be born in low TB-incidence countries, whereas young adults were more likely to be born in high-incidence countries. This trend is in keeping with UK-wide data, with 79.9% of ≥16-year-olds with TB being non-UK born compared to 60.6% for <16-year-olds.^12,13^ Sixty percent of our patient population were male, aligning with a EU/EEA study which reported that in patients above 15 years of age and below 40 years of age, there were more male than female patients with TB.^24^ However, other adolescent cohorts had a higher prevalence in females than males.^22,25^ In our cohort, the higher number of boys and young men with TB may stem from the segment of our cohort who are unaccompanied asylum-seeking minors, as most are male (89% in a recent London study^26^).

In the 2023 UKHSA report on >17-year-olds with TB, 17.1% of patients have at least one SRF from the list considered in this study.^12^ In our study, nearly a third experienced at least one of homelessness, asylum-seeking, alcohol, or drug abuse, or mental health issues. It has been estimated that 2.1% of 16–24 year-olds in London experienced homelessness between April 2022 to March 2023, compared to 16.4% of individuals in our sample.^27^ This highlights the vulnerability of this population, and the potential barriers they face to access healthcare promptly. Ten percent of the sample consisted of individuals seeking asylum, most of whom likely experienced traumatic life events, including displacement, violence, and exploitation, before arriving in the UK.^26,28^ Patients with at least one SRF were over twice as likely to receive observed therapy, highlighting the additional support already put into place to ensure these patients reach successful treatment outcomes.

Adolescents over 16 years old in the UK are predominantly managed in adult TB clinics, where follow-up may be less frequent than in paediatric clinics. Although our data did not indicate higher rates of treatment non-completion in this group compared to under-16’s in paediatric settings, one must consider the broader implications beyond these measurable outcomes. Many young adults with TB, particularly asylum-seekers, face complex physical, mental, and social health challenges, leading to difficulties in accessing services, securing good nutrition, and maintaining continuity of care when relocated. The unique clinical presentation, alongside social challenges, faced by young adults with TB underscores the need for a tailored approach to their care that may not be currently met adequately by either children or adult services. Furthermore, this study highlights the limitations of categorising those living with TB as <15 or ≥15 years old, as this approach provides little meaningful insight into the adolescent population. These findings should prompt a re-evaluation of how the WHO collects and reports global TB data to better capture the unique needs of adolescents.

Our study found that over one third of adolescent and young adult TB patients in North West London presented to secondary healthcare services more than two months after symptom onset. This is only slightly longer than median delays reported in high-incidence country literature (28-34 days)^29–31^ and shorter than a 2013 study conducted in adults in the UK (73 days, IQR 65–89).^32^ National UKHSA data for 2021–2023 highlights that those aged 15 are those least likely to experience delays in treatment; only 19.7% of this age group experienced delays of over 4 months, compared to 37.0% of those aged 45–64.^33^

**Figure 2.**
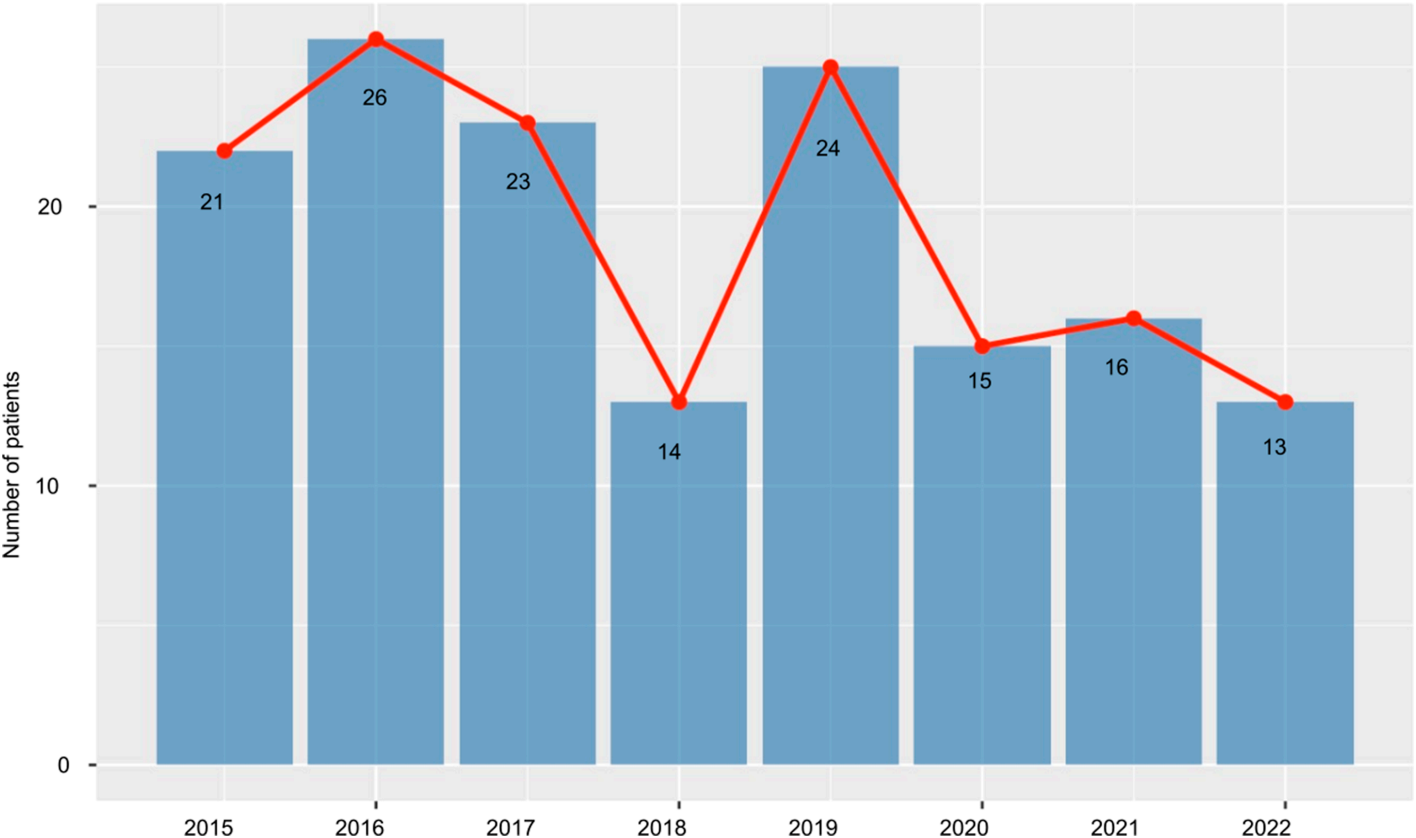
Number of TB notifications in patients 10–24 years in study centre, 2015 to 2022.

Our study period overlapped with the COVID-19 pandemic. The rate of TB notification was 22.2/year in 2015–2019, and 15.7/year in 2020–2022 (Poisson test p=0.048) see Figure 2, with slightly fewer than expected cases after the onset of the pandemic. However, no difference was observed in the post-pandemic era with regards to mean presentation delays (118 days [IQR 3–94] before 2020 and 120 days [IQR 13–78] after 2020).

Our study did not identify any statistically significant association between sex, age, SRFs and presentation delay, although the median number of days between symptoms onset and presentation were longer in patients with a history of incarceration, drug misuse and mental health issues. Previous research in adults with TB in London established that female gender, alcohol misuse and imprisonment were independently associated with presentation delay (>4 months).^34^ UKHSA data for 2021–2023 did not find a statistically significant difference in treatment delays between those with one or more SRF compared to those without any SRFs; however, this data included any age group and was not specific to children and adolescents.^33^ Our study may be underpowered to detect statistically significant associations between these factors and late presentation. It could also be that SRFs are underreported in clinical notes and therefore not captured by our data collection. Finally, it may be that these factors being analysed are not the best representative of social vulnerability in AYA, and other factors such as school attendance or living in foster care should be considered.

This study has several limitations. There were instances of missing data, particularly regarding SRFs and dates of symptom onset: as the latter were estimated based on patient report at the first point of contact with secondary healthcare, there could also be inaccuracies due to recall bias. Despite our patients originating from North West London, a region of relatively high TB incidence, TB in young people remains a rare condition, and our sample size is small. The lack of statistically significant findings must thus be interpreted with caution as they could be due to type 2 error. As a single-centre study, our findings may not be generalisable to other regions within the UK or to other high-income, low-prevalence countries with different demographic and socioeconomic profiles. Future research should aim to include multiple centres and more diverse populations to ensure broader applicability.

Overall, our study adds insight into the distinct characteristics and healthcare needs of AYA with TB in a high-income, low TB-prevalence setting. We found that nearly half of patients with PTB have cavitary disease, and a third have one or more SRFs. By focusing on the challenges adolescents with TB face, this study underscores the importance of developing adolescent-focused TB care models that prioritise both physical and mental health outcomes. Such models could serve as a blueprint for improving TB care for young people in other high-income, low-prevalence settings.
